# Calycosin attenuates *Angiostrongylus cantonensis*-induced parasitic meningitis through modulation of HO-1 and NF-*κ*B activation

**DOI:** 10.1017/S0031182022001408

**Published:** 2023-04

**Authors:** Cheng-You Lu, Ke-Min Chen, Wei-Wen Kuo, Shih-Chan Lai, Tsung-Jung Ho, Po-Tang Lai, Chih-Yang Huang, Tso-Fu Wang

**Affiliations:** 1 Department of Post-Baccalaureate Medicine, College of Medicine, National Chung Hsing University, Taichung, Taiwan; 2 Department of Parasitology, School of Medicine, Chung Shan Medical University, Taichung, Taiwan; 3 Department of Biological Science and Technology, China Medical University, Taichung, Taiwan; 4 Ph.D. Program for Biotechnology Industry, China Medical University, Taichung, Taiwan; 5 Clinical Laboratory, Chung Shan Medical University Hospital, Taichung, Taiwan; 6 Integration Center of Traditional Chinese and Modern Medicine, Hualien Tzu Chi Hospital, Hualien, Taiwan; 7 Department of Chinese Medicine, Hualien Tzu Chi Hospital, Buddhist Tzu Chi Medical Foundation, Tzu Chi University, Hualien, Taiwan; 8 School of Post Baccalaureate Chinese Medicine, College of Medicine, Tzu Chi University, Hualien, Taiwan; 9 Division of Endodontics and Periodontology, Department of Stomatology, Taipei Veterans General Hospital, Taipei, Taiwan; 10 Department of Dentistry, Hualien Tzu Chi Hospital, Buddhist Tzu Chi Medical Foundation, Hualien, Taiwan; 11 Cardiovascular and Mitochondrial Related Disease Research Center, Hualien Tzu Chi Hospital, Buddhist Tzu Chi Medical Foundation, Hualien, Taiwan; 12 Graduate Institute of Biomedical Sciences, China Medical University, Taichung, Taiwan; 13 Department of Biological Science and Technology, Asia University, Taichung, Taiwan; 14 Center of General Education, Buddhist Tzu Chi Medical Foundation, Tzu Chi University of Science and Technology, Hualien, Taiwan; 15 Department of Medical Research, China Medical University Hospital, China Medical University, Taichung, Taiwan; 16 Department of Hematology and Oncology, Hualien Tzu Chi Hospital, Buddhist Tzu Chi Medical Foundation, Hualien, Taiwan; 17 College of Medicine, Tzu-Chi University, Hualien, Taiwan

**Keywords:** *Angiostrongylus cantonensis*, anti-inflammation, calycosin, eosinophilic meningitis, HO-1

## Abstract

*Angiostrongylus cantonensis* causes a form of parasitic meningitis in humans. Albendazole (ABZ) kills nematode larvae in the brain. However, dead larvae can trigger a severe inflammatory response, resulting in brain damage. Accumulating evidence suggests that calycosin represents a potential anti-inflammatory therapeutic candidate. In this study, we investigated the combined effects of ABZ and calycosin in angiostrongyliasis caused by *A. cantonensis* in BALB/c mice. Inflammatory mediators (such as phospho-nuclear factor-*κ*B, cyclooxygenase-2, matrix metalloproteinase-9, tumour necrosis factor-*α* and interleukin-1*β*) are associated with the development of meningitis and immune inflammatory reactions. We found that *A. cantonensis* significantly induces inflammatory mediator production and increases the blood–brain barrier (BBB) permeability. However, co-administration of both ABZ and calycosin markedly suppressed meningitis and inflammatory mediator production and decreased the BBB permeability compared to treatment with a single drug. Furthermore, calycosin and ABZ plus calycosin treatment facilitated production of the antioxidant haem oxygenase-1 (HO-1). Moreover, co-therapy with ABZ and calycosin failed to mitigate angiostrongyliasis in the presence of tin-protoporphyrin IX, an HO-1-specific inhibitor. This finding suggests that the beneficial effects of ABZ plus calycosin treatment on the regulation of inflammation are mediated by the modulation of HO-1 activation. The present results provide new insights into the treatment of human angiostrongyliasis using co-therapy with ABZ and calycosin.

## Graphical abstract

**Figure:**
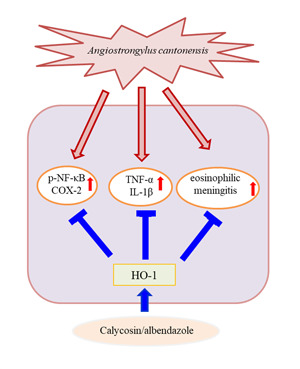

## Introduction

The mature adults of *Angiostrongylus cantonensis* are zoonotic nematodes that thrive in the pulmonary arteries of rats. Non-permissive hosts, such as humans, may unintentionally ingest third-stage (L3) nematode larvae through food, such as snails, slugs or raw or undercooked vegetables (Alto, 2001). *Angiostrongylus cantonensis* causes angiostrongyliasis, which is characterized by severe central nervous system (CNS) inflammation, eosinophilic meningitis and eosinophilic meningoencephalitis (Hsu *et al*., 1990; Ismail and Arsura, 1993; Alto, 2001). The parasite has been found to infect humans and other mammals, with a wide and ever-increasing distribution across regions such as East Asia, Southeast Asia, the Pacific Islands and the Caribbean (Wang *et al*., 2018). Worldwide, *A. cantonensis* infection cases occur every year. According to a review published in 2008, nearly 3000 cases of human angiostrongyliasis have been documented worldwide (Wang *et al*., 2008). However, this number has risen rapidly in recent years. In a prospective descriptive study conducted from June 2008 to January 2014 in a Vietnamese hospital, *A. cantonensis* was found to be an important cause of eosinophilic meningitis, accounting for 67.3% (37/55) of the cases (McBride *et al*., 2017). These outbreaks have caused great concern regarding the treatment for *A. cantonensis* infection among the general public. Therefore, research on *A. cantonensis* is crucial and of significant socioeconomic importance globally. Thus, identifying a new strategy to suppress *A. cantonensis*-mediated CNS inflammation, eosinophilic meningitis and eosinophilic meningoencephalitis is critical.

Many inflammatory mediators are linked to several inflammatory diseases, including CNS inflammation. Previous studies have demonstrated that the inflammatory mediators cyclooxygenase-2 (COX-2) (Crofford, 1997), tumour necrosis factor-*α* (TNF-*α*) (Vassalli, 1992), interleukin-1*β* (IL-1*β*) (McAfoose and Baune, 2009), matrix metalloproteinase-9 (MMP-9) (Stamenkovic, 2003) and nuclear factor (NF)-*κ*B (Kaltschmidt *et al*., 2005) are expressed at low levels under normal physiological conditions and are highly induced in response to inflammation or pathological processes. Moreover, an increasing number of studies have revealed that TNF-*α*, IL-1*β* (Tu and Lai, 2006), MMP-9 (Chen *et al*., 2004) and NF-*κ*B (Chiu and Lai, 2013) may participate in the pathogenesis of CNS inflammation during *A. cantonensis* infection.

Calycosin represents the major isoflavonoid in Huang Qi (Radix Astragali Mongolici), a traditional Chinese herbal medicine (Li *et al*., 2011). Calycosin can exhibit anti-inflammatory mediator- or cytokine-like activity, including decrease in the COX-2, IL-1*β* and TNF-*α*, as well as mediates NF-*κ*B signalling (Hoo *et al*., 2010; Su *et al*., 2016; Dong *et al*., 2018). However, no study has delineated the potential of calycosin in *A. cantonensis*-induced CNS inflammation. With this in mind, the current study was carried out to explore whether calycosin could ameliorate *A. cantonensis-*induced CNS inflammation and eosinophilic meningitis and thereby ascertain the underlying mechanisms.

## Materials and methods

### Chemical reagents and antibodies

The antibodies used in this study were anti-MMP-9, anti-phospho-NF-*κ*B (p-P65), anti-COX-2 and anti-*β*-actin (Santa Cruz Biotechnology Inc., CA, USA). Haem oxygenase-1 (HO-1) was obtained from Abclonal Company, Inc. (MA, USA). Albendazole (ABZ), an anthelmintic or anti-worm medication, and calycosin were purchased from Sigma-Aldrich (St. Louis, MO, USA). Tin-protoporphyrin IX (SnPPIX) was purchased from Cayman Chemical (Ann Arbor, Michigan, USA). ABZ was dissolved in a normal saline solution. Calycosin and SnPPIX were dissolved in dimethyl sulphoxide and administered to the animals at a final concentration of <0.1%.

### Experimental animals

We used 5-week-old male BALB/c mice to establish the *A. cantonensis*-infected mouse model. The mice were purchased from the National Laboratory Animal Center (Taipei, Taiwan) and housed under a 12 h light and dark cycle with free access to water and food.

### Animal infection protocol

The third-stage larvae (L3, infective larvae) of *A. cantonensis* were obtained from naturally infected giant African snails (*Achatina fulica*) that were purchased from Heping District (Taichung, Taiwan) (Chin *et al*., 2018). The larvae were liberated from the minced snail tissues by pepsin (Sigma, USA) digestion. The identity of the L3 larvae of *A. cantonensis* was confirmed as described earlier (Ash, 1970). To assess whether the larvae found were *A. cantonensis*, we fed them to rats and then examined the rat brains after 2–3 weeks for evidence of infection. In this study, food and water were prohibited for 12 h before infection. Thirty male BALB/c mice were randomly allocated to 6 groups (control, and days 6, 12, 18, 24 and 30) of 5 mice each. Mice in the 5 experimental groups (days 6, 12, 18, 24 and 30) were infected with 50 *A. cantonensis* larvae by oral inoculation and were sacrificed on days 6, 12, 18, 24 or 30 post-infection (PI). Control mice received only water and were euthanized on day 30 PI.

### Animal treatment

Twenty mice were randomly divided into 4 treatment groups (5 mice per group). The 4 groups were treated with ABZ (10 mg kg^−1^ day^−1^, oral administration), calycosin (30 mg kg^−1^ day^−1^, intraperitoneal administration), ABZ (10 mg kg^−1^ day^−1^, oral administration) combined with calycosin (30 mg kg^−1^ day^−1^, intraperitoneal administration) and SnPPIX (15 mg kg^−1^ day^−1^, intraperitoneal administration) combined with ABZ (10 mg kg^−1^ day^−1^, oral administration) and calycosin (30 mg kg^−1^ day^−1^, intraperitoneal administration), respectively, for 19 consecutive days. Drug administration was initiated on days 6–24 after infection. All mice were killed 25 days after inoculation.

### Brain and blood sample collection

The brains were dissected, placed in powdered dry ice and stored at −80 °C. Coronal sections (20 *μ*m) at the level of the striatum were cut on a cryostat at −18 °C, collected on glass slides coated with Vectabond (Vector Labs, Newark, CA, United States) and stored at −80 °C until immunostaining. All brain tissue extracts from each group were obtained by homogenizing in a lysis buffer (0.05 m Tris-HCl, pH 7.4, 0.15 m NaCl, 0.25% deoxycholic acid, 1% NP-40, 1 mm EDTA) containing the following protease inhibitors: 0.1 mm PMSF, 10 *μ*m sodium orthovanadate and 20 *μ*g mL^−1^ leupeptin at a ratio of 100 mg tissue mL^−1^ lysis buffer. The homogenates were placed on ice and centrifuged at 10 000 ***g*** (for 30 min at 4 °C). The supernatants were collected and stored at −80 °C for further experiments.

### Western blotting

Western blotting analyses were carried out as previously described, with slight modifications (Chin *et al*., 2018; Lin *et al*., 2019; Liu *et al*., 2020; Chang *et al*., 2021). Protein concentrations in the homogenates were then determined using the Bradford assay (Bio-Rad, Hercules, CA, USA). Thereafter, the protein samples were separated by 10% sodium dodecyl sulphate-polyacrylamide gel electrophoresis. The resolved proteins were transferred to polyvinylidene fluoride membranes (Merck Millipore, MA, USA). The membranes were blocked with 5% defatted milk in phosphate-buffered saline (PBS) (pH 7.4) and then exposed to the appropriate antibodies. All bands were visualized with horseradish peroxidase-conjugated secondary antibodies (Santa Cruz Biotechnology, California, USA) using an enhanced chemiluminescence system (Merck Millipore, MA, USA).

### Enzyme-linked immunosorbent assay

TNF-*α* and IL-1*β* levels were measured using TNF-*α* (ab100785) and IL-1*β* (ab100768) enzyme-linked immunosorbent assay (ELISA) kits (Abcam, MA, USA), respectively, in accordance with the manufacturer's protocol. Fluorescence was measured on a microplate reader at excitation/emission wavelengths of 488/535 nm. ELISA was performed as described previously with slight modifications (Lu *et al*., 2022).

### Worm recovery

For larval recovery, the brain of each mouse was dissected into small pieces and homogenized separately in 15 mL of 0.25% sodium citrate in PBS, followed by centrifugation (1400 ***g***, 10 min). Larvae were counted by visualizing at 25× magnification using a dissection microscope as described previously (Chen *et al*., 2022).

### Histology

Mouse brains were immediately removed and fixed in 10% neutral-buffered formalin for 24 h. The fixed brains were dehydrated in a graded ethanol series (50, 75, 95 and 100%), replaced with xylene, and embedded in paraffin at 55 °C for 24 h. Several serial sections were cut at 10 *μ*m thickness and stained with haematoxylin and eosin (Muto, Japan). Pathological changes were examined under a microscope (CKX53; Olympus, Tokyo, Japan).

### Blood–brain barrier permeability assay

Two hours before sacrifice, mice were injected with 2% Evans blue solution prepared in saline (100 mg kg^−1^ body weight; Sigma, St. Louis, MO, USA) into the tail vein. The concentration of Evans blue in the brain was determined as described previously, with slight modifications (Chiu and Lai, 2013), to assess blood–brain barrier (BBB) permeability. The average concentration of Evans blue in the cerebrospinal fluid (CSF) was calculated by measuring absorbance of the CSF at 620 nm using a spectrophotometer (Hitachi U3000; Tokyo, Japan).

### Statistical analysis

Statistical analyses were performed by multiple comparisons that were accessed through one-way analysis of variance and using SigmaPlot software (version 10.0; Systat Software Inc., San Jose, CA, USA) with GraphPad Prism 8. Comparisons between 2 groups were performed using the Student's *t*-test. In all tests, a value of *P < 0.05 was considered statistically significant, while **P < 0.01 and ***P < 0.001 indicated increased statistical significance.

## Results

### Time-course studies of MMP-9, COX-2, p-NF-*κ*B and HO-1 levels from the brains of mice infected with *A. cantonensis*

**Fig. 1.**
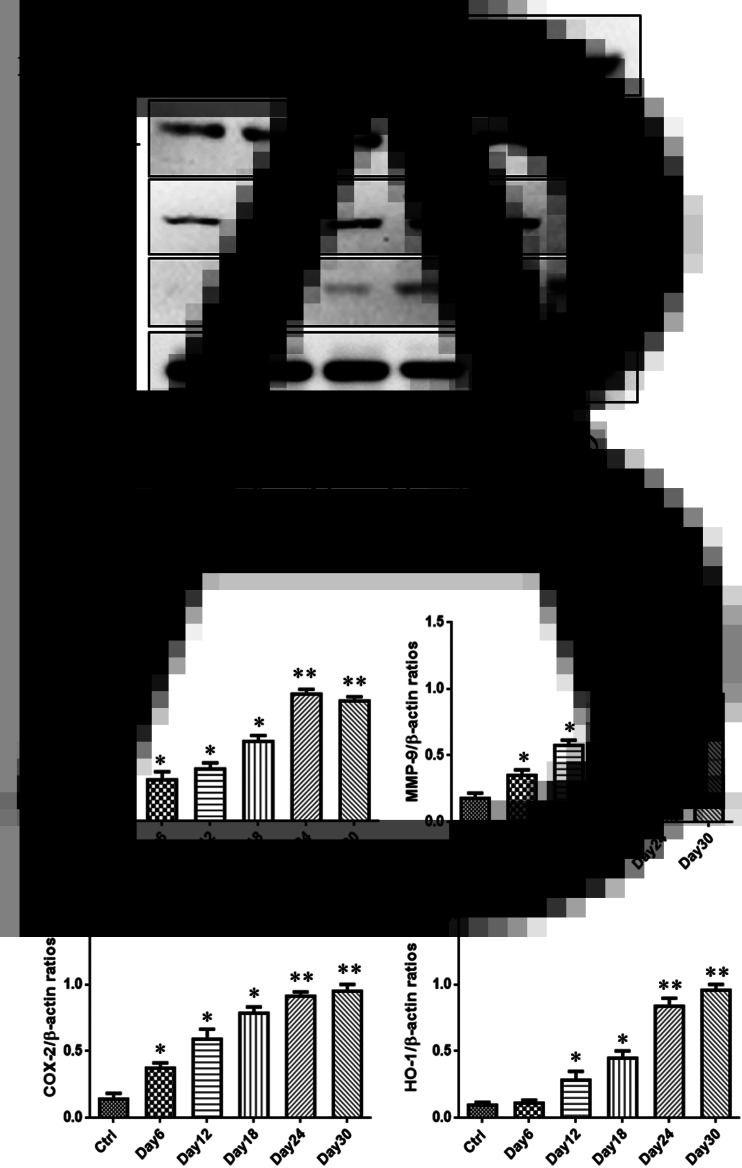
Protein levels of p-NF-*κ*B, MMP-9, COX-2 and HO-1 in the brains of mice infected with *Angiostrongylus cantonensis*. (A) p-NF-*κ*B, MMP-9, COX-2 and HO-1 bands were detected at all time points. (B) *P < 0.05 and **P < 0.01 *vs* day 0 group. Data are presented as mean ± s.d. of 3 independent experiments.

The inflammatory mediators MMP-9, COX-2 and p-NF-*κ*B are associated with brain inflammation. Therefore, we examined the protein levels of MMP-9, COX-2 and p-NF-*κ*B after *A. cantonensis* infection in a time-dependent manner. Time-course studies for MMP-9 level showed significant increases (*P* < 0.05) from day 10 to day 25. Furthermore, COX-2 and p-NF-*κ*B levels significantly increased (*P* < 0.05) from day 6 to day 30 (Fig. 1A).

HO-1 is a cytoprotective enzyme that responds to oxidative and inflammatory stimuli. Therefore, we examined the expression of HO-1 after *A. cantonensis* infection in a time-dependent manner through western blotting. HO-1 levels were significantly increased (*P* < 0.05) from day 6 to day 30 (Fig. 1B). The results from 3 repeated and separate experiments were similar.

### Effects of ABZ combined with calycosin from the brain of mice infected with *A. cantonensis* infection

**Fig. 2.**
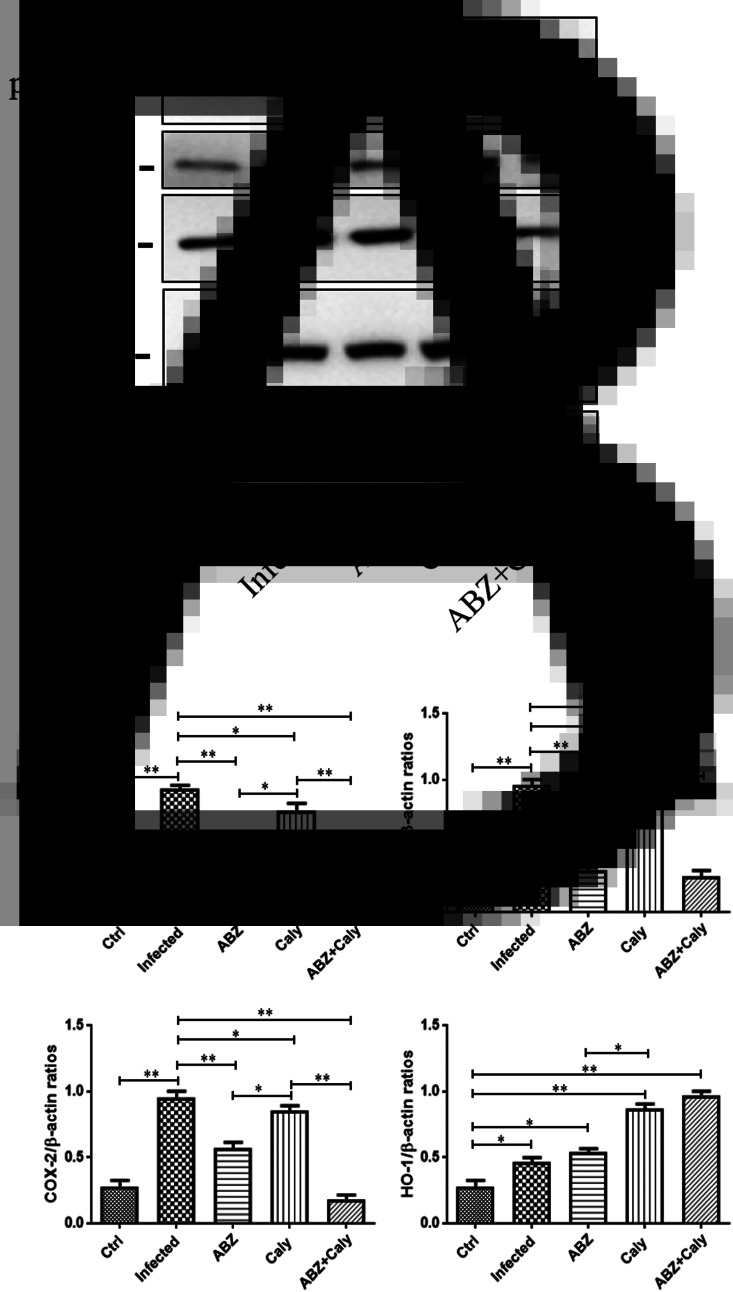
Protein levels of p-NF-*κ*B, MMP-9, COX-2 and HO-1 in the brain of mice. (A) p-NF-*κ*B, MMP-9, COX-2 and HO-1 bands were detected for all treatment groups. (B) *P < 0.05 and **P < 0.01 indicate the significant difference. Data are presented as mean ± s.d. of 3 independent experiments. Ctrl, control group; Infected, *A. cantonensis* infection group; ABZ, albendazole treatment group; Caly, calycosin treatment group; ABZ + Caly, albendazole combined with calycosin treatment group.

To assess the effects of ABZ, calycosin and ABZ combined with calycosin treatment, we detected changes in the protein levels of MMP-9, COX-2 and p-NF-*κ*B using western blotting. The results showed that MMP-9, COX-2 and p-NF-*κ*B levels were significantly increased in the infection groups compared to the control group. Nevertheless, MMP-9, COX-2 and p-NF-*κ*B levels were significantly lower in the ABZ, calycosin and ABZ combined with calycosin treatment groups, particularly in the ABZ combined with calycosin treatment group, compared with the *A. cantonensis*-infected mice (Fig. 2A and B). Moreover, HO-1 expression was moderately increased in the infection and ABZ-only treatment groups compared to the control and significantly increased under calycosin or ABZ combined with calycosin treatment compared to the control, infection and ABZ-only treatment groups (Fig. 2C). The results from 3 repeated and separate experiments were similar.

### Changes in larvae recovery, Evans blue units, TNF-*α* and IL-1*β* from the brain of mice treated with ABZ alone or ABZ combined with calycosin caused by *A. cantonensis* infection

**Fig. 3.**
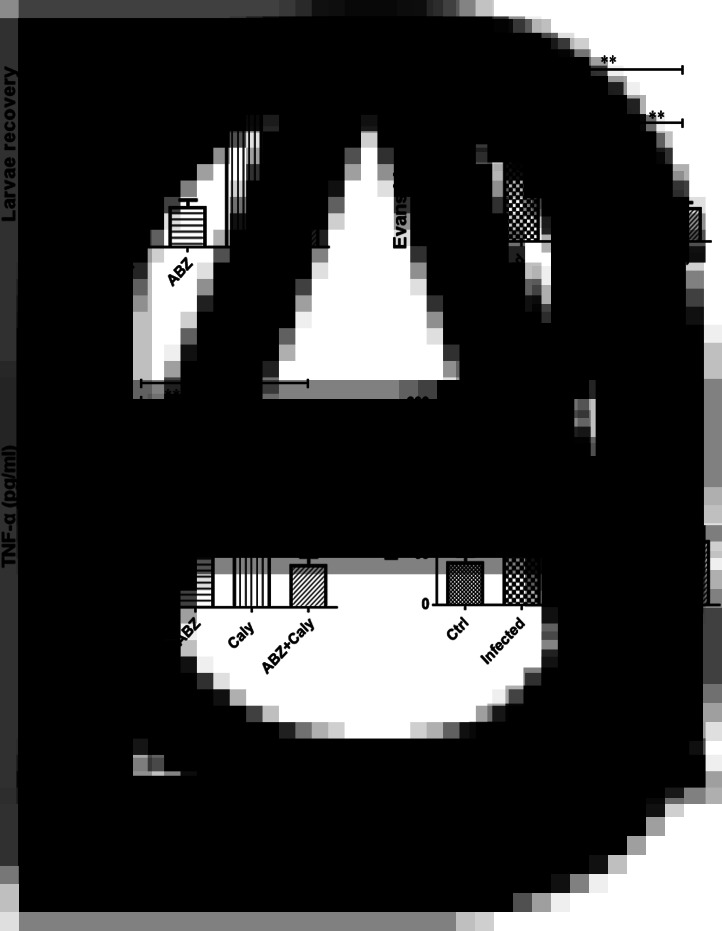
Levels of larval recovery, Evans blue, TNF-*α* and IL-1*β*. Larval recovery (A), Evans blue (B), TNF-*α* (C) and IL-1*β* (D) were detected in all treatment groups. *P < 0.05, **P < 0.01 and ***P < 0.001 indicate the significant difference. Data are presented as mean ± s.d. of 3 independent experiments. Ctrl, control group; Infected, *A. cantonensis* infection group; ABZ, albendazole treatment group; Caly, calycosin treatment group; ABZ + Caly, albendazole combined with calycosin treatment group.

Larval recovery was significantly increased in infected mice treated with calycosin alone but decreased in ABZ-alone or ABZ–calycosin co-treatment (Fig. 3A). Moreover, BBB permeability was enhanced in mice with eosinophilic meningitis or meningoencephalitis, which may result from *A. cantonensis* infection, and was detected by performing Evans blue extravasation assay during *A. cantonensis* infection (Fig. 3B). Additionally, TNF-*α* and IL-1*β* are key proinflammatory cytokines in inflammatory diseases (Turner *et al*., 2014). Therefore, we also assessed the levels of TNF-*α* (Fig. 3C) and IL-1*β* (Fig. 3D) in a time-dependent manner, following *A. cantonensis* infection, using ELISA. The results demonstrated that Evans blue, TNF-*α* and IL-1*β* levels were significantly increased in the infection groups compared to the control group. Moreover, Evans blue, TNF-*α* and IL-1*β* levels were significantly decreased in the ABZ, calycosin and ABZ plus calycosin treatment groups, especially in the ABZ plus calycosin treatment group, compared with the *A. cantonensis*-infected mice. The results from 3 repeated and separate experiments were consistent.

### Changes in MMP-9, COX-2, p-NF-*κ*B, HO-1 and *β*-actin protein levels in the brains of mice treated with ABZ combined with calycosin or SnPPIX caused by *A. cantonensis* infection

**Fig. 4.**
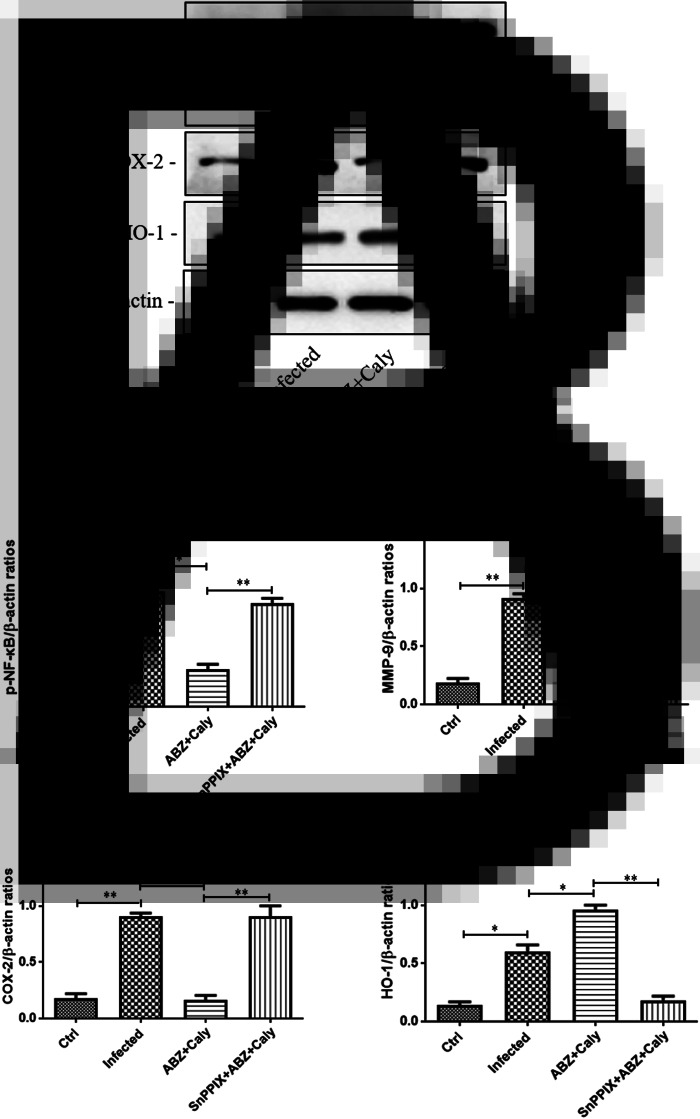
Protein levels of p-NF-*κ*B, MMP-9, COX-2 and HO-1 in the brain of mice. (A) p-NF-*κ*B, MMP-9, COX-2 and HO-1 bands were detected for all treatment groups. (B) *P < 0.05 and **P < 0.01 indicate the significant difference. Data are presented as mean ± s.d. of 3 independent experiments. Ctrl, control group; Infected, *A. cantonensis* infection group; ABZ + Caly, albendazole combined with calycosin treatment group; SnPPIX + ABZ + Caly, SnPPIX and albendazole combined with calycosin treatment group.

To confirm the protective effects of HO-1, we performed additional experiments to assess the effect of SnPPIX, a potent competitive inhibitor of HO-1 (Hyvelin *et al*., 2010). The protein levels of MMP-9, COX-2 and p-NF-*κ*B in the treated groups were similar to those observed in Fig. 2. The levels of MMP-9, COX-2 and p-NF-*κ*B were significantly increased and HO-1 levels were moderately increased in the infection groups; however, MMP-9, COX-2 and p-NF-*κ*B levels were significantly decreased and HO-1 levels were significantly increased in the ABZ plus calycosin treatment groups. Additionally, co-treatment with SnPPIX, ABZ and calycosin reversed the effects of ABZ plus calycosin treatment, i.e. increased MMP-9, COX-2 or p-NF-*κ*B and decreased HO-1 expression (Fig. 4). The results from 3 repeated and separate experiments were similar.

### Changes in larval recovery, Evans blue, TNF-*α* and IL-1*β* levels after treatment with ABZ alone or ABZ–calycosin in the brains of mice during *A. cantonensis* infection

To confirm the inhibitory effect of SnPPIX on HO-1 activity, we assessed the larval recovery, Evans blue, TNF-*α* and IL-1*β* levels. Larval recovery was significantly increased in infected mice treated with calycosin alone but decreased in ABZ-alone or ABZ–calycosin co-treatment groups.

**Fig. 5.**
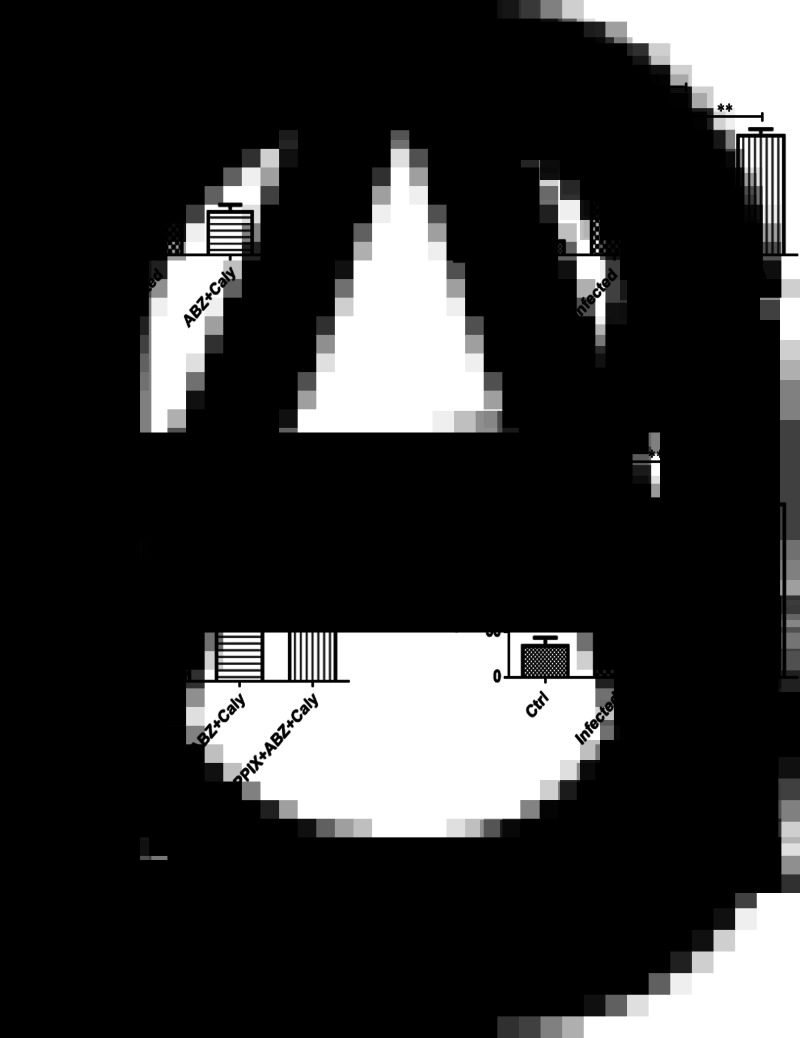
Levels of larval recovery, Evans blue, TNF-*α* and IL-1*β*. Larval recovery (A), Evans blue (B), TNF-*α* (C) and IL-1*β* (D) were detected for all treatment groups. *P < 0.05 and **P < 0.01 indicate the significant difference. Data are presented as mean ± s.d. of 3 independent experiments. Ctrl, control group; Infected, *A. cantonensis* infection group; ABZ, albendazole treatment group; Caly, calycosin treatment group; ABZ + Caly, albendazole combined with calycosin treatment group.

In contrast, larval recovery was decreased by ABZ–calycosin or SnPPIX–ABZ–calycosin co-treatment (Fig. 5A). The Evans blue units (Fig. 5B), TNF-*α* (Fig. 5C) and IL-1*β* (Fig. 5D) levels were significantly increased in the infection groups compared with the control group; however, their levels were significantly decreased in the ABZ plus calycosin treatment group. Furthermore, co-treatment with SnPPIX, ABZ and calycosin reversed the effects of ABZ and calycosin. The results from the 3 repeated and separate experiments were similar.

### Histopathological examinations

**Fig. 6.**
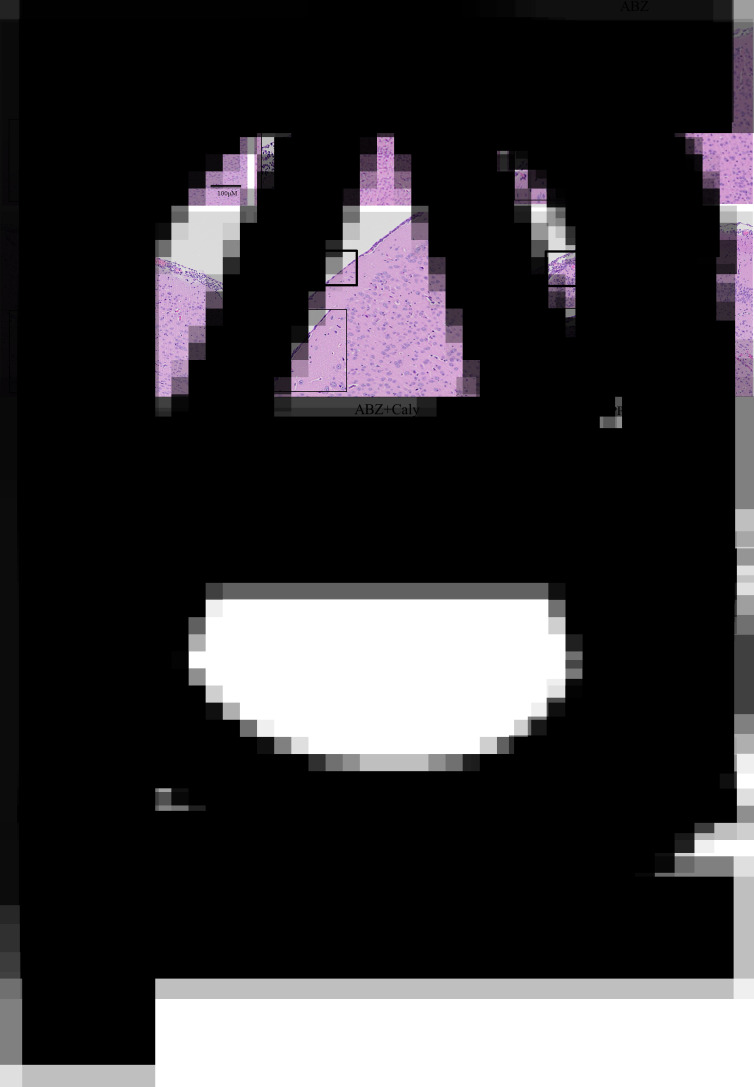
Pathological morphology of the subarachnoid space in mice evaluated using haematoxylin and eosin staining. (A) Control group (Ctrl). (B) *Angiostrongylus cantonensis* infection group (Infected). (C) Albendazole treatment group (ABZ). (D) Calycosin treatment group (Caly). (E) Albendazole combined with calycosin treatment group (ABZ + Caly). (F) SnPPIX and albendazole combined with calycosin treatment group (SnPPIX + ABZ + Caly).

Optical microscopic examination of tissues stained with haematoxylin and eosin showed that eosinophilic meningitis was induced in the infected groups. The results demonstrated severe haemorrhage, severe thickening of the meninges and large-scale infiltration of the subarachnoid space by leucocytes in *A. cantonensis*-infected mouse brain tissues compared to normal controls. Haemorrhage, meningeal thickness and leucocyte infiltration were moderately reduced by the individual treatment with ABZ or calycosin. However, ABZ in combination with calycosin showed a marked reduction in haemorrhage, meningeal thickness and leucocyte number. In addition, co-treatment with SnPPIX, ABZ and calycosin reversed the effects of ABZ and calycosin (Fig. 6).

## Discussion

*Angiostrongylus cantonensis* causes eosinophilic meningitis in mice that attains a peak at approximately 3 weeks. In parallel with this pathogenesis, infected mice show signs of a gradual increase in inflammation, attaining a peak at the same time (Sugaya and Yoshimura, 1988; Sasaki *et al*., 1993). Previous studies have shown that TNF-*α*, IL-1*β* (Tu and Lai, 2006), MMP-9 (Chen *et al*., 2004), COX-2 (Chen *et al*., 2021) and NF-*κ*B (Chiu and Lai, 2013) may participate in the pathogenesis of CNS inflammation during *A. cantonensis* infection. In this study, significant increases in TNF-*α*, IL-1*β*, MMP-9, COX-2 and p-NF-*κ*B levels in brain samples from mice infected with *A. cantonensis* were demonstrated in a time-dependent manner. In contrast, levels of these inflammatory enzymes decreased in response to treatment with ABZ, a broad-spectrum anthelmintic.

Calycosin is an isoflavonoid and a major bioactive chemical in Huang Qi (Li *et al*., 2011). Furthermore, calycosin can exert neuroprotective and anti-inflammatory effects (Su *et al*., 2016; Lu *et al*., 2022) and reduce cellular oxidative damage (Guo *et al*., 2002; Lu *et al*., 2022). Likewise, our results revealed that calycosin protected mice against *A. cantonensis*-induced inflammation and reduced the production of inflammatory enzymes. However, its therapeutic effects could not completely suppress the *A. cantonensis*-induced inflammation, probably owing to the persistence of the parasites even after treatment.

ABZ kills parasites such as the nematode *A. cantonensis* by blocking the absorption of glucose by the larvae (Hwang and Chen, 1988; Lakwo *et al*., 1998). Thus far, the drug has shown good results for the treatment of angiostrongyliasis (Hwang and Chen, 1991). ABZ exhibits marked larvicidal activity against angiostrongyliasis. However, certain studies have revealed that ABZ and mebendazole are not recommended for angiostrongyliasis treatment because they may exacerbate the neurological symptoms as a side-effect (Hidelaratchi *et al*., 2005; Wang *et al*., 2006; Wan *et al*., 2018). Additionally, treatment with ABZ alone in eosinophilic meningitis cannot completely inhibit the inflammatory reaction (Lan *et al*., 2004). Thus, treatment usually involves co-administration of corticosteroids to limit the inflammatory reaction (Chotmongkol *et al*., 2006, 2009; Diao *et al*., 2011). Corticosteroids have been used for a long time in the clinic and have played a useful role in suppressing inflammation in the brain. However, steroids have side-effects such as infection (immunodepression), gastrointestinal symptoms, osteoporosis, weight gain and steroid withdrawal syndrome (Prociv and Turner, 2018; McAuliffe *et al*., 2019). To increase the survival rate and quality of treatment, it may be helpful to replace steroids with other anti-neuroinflammatory agents. Therefore, the present study focused on the evaluation of calycosin. The application of combination therapy with ABZ and calycosin is a prudent course of action. This combination therapy effectively suppressed excessive inflammation compared to treatment with calycosin or ABZ alone.

HO represents a class of microsomal enzymes that includes HO-1, HO-2 and HO-3. HO degrades the prooxidant haem to carbon monoxide, biliverdin (subsequently reduced to bilirubin) and ferrous iron (Maines, 1997; Turkseven *et al*., 2005). HO-1 activity is significantly induced by numerous stimuli, including haem, heavy metals, hormones, oxidative stress (Platt and Nath, 1998; Novotny and Vitek, 2003; Lu *et al*., 2022) and traumatic brain injury (Okubo *et al*., 2013). HO-1 induction has been shown to confer protection, whereas its abrogation has been revealed to accelerate cellular injuries (Akagi *et al*., 2002). Additionally, HO-1 modulates brain inflammation and apoptosis in mice with angiostrongyliasis (Chen *et al*., 2022). Our results indicated that HO-1 level was slightly increased in the *A. cantonensis* infection and ABZ treatment groups. Moreover, co-treatment with ABZ and calycosin suppressed the expression of inflammatory cytokines and *A. cantonensis*-induced inflammation and significantly upregulated HO-1 expression, indicating that HO-1 may play a crucial role in the progression of *A. cantonensis*-induced inflammation. To better understand the role of HO-1 induced by calycosin, *A. cantonensis*-infected mice were pre-treated with SnPPIX, a well-characterized HO-1 inhibitor. Our results demonstrated that, with the combination of SnPPIX and ABZ–calycosin treatment, SnPPIX reversed the ABZ–calycosin-induced upregulation of expression of HO-1 and inflammatory cytokines in *A. cantonensis*-infected mice. This finding suggests that calycosin may act as an HO-1 activator that upregulates and maintains HO-1 expression after *A. cantonensis* infection. These results indicate that modulation of HO-1 and NF-*κ*B activation after calycosin treatment protects against inflammation in *A. cantonensis*-infected mice. However, the role of calycosin in *A. cantonensis*-infected mice remains unclear. As the current study was limited to *in vivo* systems, future work is required to evaluate the *in vitro* effects and molecular mechanisms of calycosin.

## Conclusions

Our study is the first to show that calycosin exerts anti-inflammatory effects in *A. cantonensis*-infected mice. The results provide evidence that ABZ–calycosin co-treatment effectively suppresses inflammatory mediator production and eosinophilic meningitis through the modulation of HO-1 and NF-*κ*B activity, suggesting that the combination therapy with ABZ and calycosin may also reduce the side-effects of ABZ. Our study lays forth a probable explanation for the beneficial effect of calycosin in the prevention of eosinophilic meningitis caused by *A. cantonensis*. This study was limited to the finding that calycosin attenuates *A. cantonensis*-induced parasitic meningitis through modulation of HO-1 and NF-*κ*B activation. Future work is required to evaluate the detailed underlying molecular mechanisms linked to the therapeutic efficacy of combination therapy to improve anti-parasitic meningitis effects.

## Data Availability

The data and material of this study are available for publishing in public.
